# SOCS1 and SOCS3 as key checkpoint molecules in the immune responses associated to skin inflammation and malignant transformation

**DOI:** 10.3389/fimmu.2024.1393799

**Published:** 2024-06-21

**Authors:** Martina Morelli, Stefania Madonna, Cristina Albanesi

**Affiliations:** Laboratory of Experimental Immunology, Istituto Dermopatico dell'Immacolata - Istituto di Ricovero e Cura a Carattere Scientifico (IDI-IRCCS), Rome, Italy

**Keywords:** suppressors of cytokine signaling (SOCS), Janus Activated Kinases (JAK), skin inflammation, immunemediated skin diseases, skin cancer, JAK inhibitors

## Abstract

SOCS are a family of negative inhibitors of the molecular cascades induced by cytokines, growth factors and hormones. At molecular level, SOCS proteins inhibit the kinase activity of specific sets of receptor-associated Janus Activated Kinases (JAKs), thereby suppressing the propagation of intracellular signals. Of the eight known members, SOCS1 and SOCS3 inhibit activity of JAKs mainly induced by cytokines and can play key roles in regulation of inflammatory and immune responses. SOCS1 and SOCS3 are the most well-characterized SOCS members in skin inflammatory diseases, where their inhibitory activity on cytokine activated JAKs and consequent anti-inflammatory action has been widely investigated in epidermal keratinocytes. Structurally, SOCS1 and SOCS3 share the presence of a N-terminal domain containing a kinase inhibitory region (KIR) motif able to act as a pseudo-substrate for JAK and to inhibit its activity. During the last decades, the design and employment of SOCS1 and SOCS3-derived peptides mimicking KIR domains in experimental models of dermatoses definitively established a strong anti-inflammatory and ameliorative impact of JAK inhibition on skin inflammatory responses. Herein, we discuss the importance of the findings collected in the past on SOCS1 and SOCS3 function in the inflammatory responses associated to skin immune-mediated diseases and malignancies, for the development of the JAK inhibitor drugs. Among them, different JAK inhibitors have been introduced in the clinical practice for treatment of atopic dermatitis and psoriasis, and others are being investigated for skin diseases like alopecia areata and vitiligo.

## 1 Introduction

SOCS1 and SOCS3 are proteins that play crucial roles in the regulation of JAK/STAT signaling pathways (1). SOCS1 and SOCS3 belong to SOCS protein family, comprising eight family members: SOCS1–SOCS7 and the cytokine-inducible SH2-domain-containing protein (CIS). All SOCS proteins show conserved structural similarities and mechanisms of action but with unique aspects for different family members (1, 2). Both SOCS1 and SOCS3 control the intensity and duration of immune responses and prevent uncontrolled inflammation. In particular, SOCS1 and SOCS3 are most closely involved in the regulation of the effects induced by IL-4, IL-6, and IFN-γ, which influence the polarization of lymphocytes and the activation of myeloid cells (2).

SOCS1 and SOCS3 dysregulation has been linked to various pathological conditions, making them potential targets for therapeutic interventions in diseases characterized by excessive immune responses and inflammatory cytokine stimulation (2). For example, reduced expression of SOCS1 has been associated with autoimmune diseases such as rheumatoid arthritis and systemic lupus erithematosus. SOCS1 has also implications in cancer, where its dysregulation can contribute to abnormal immune responses (3). Dysregulation of SOCS3 has been associated with various inflammatory conditions, including rheumatoid arthritis, inflammatory bowel disease, and psoriasis (4). As for SOCS1, aberrant expression of SOCS3 has been observed in certain cancers, influencing the tumor microenvironment and immune responses (5).

There is considerable evidence that SOCS1 and SOCS3 play important roles in viral immune evasion involving a broad range of viruses. In fact, SOCS1 and SOCS3 are broadly hijacked by viruses to function effectively as viral virulence factors (6).

In this review, we will retrace the numerous studies obtained during the last two decades and aimed at deciphering the structure and function of SOCS1 and SOCS3 in the inflammatory contexts associated to skin immune-mediated diseases and malignancies. We discussed on how this investigative studies have led to the design and development of peptides mimicking SOCS1 and SOCS3 inhibitory activity, and to the formulation of small molecules inhibiting JAKs as novel therapeutics for skin dermatoses.

## 2 Mechanisms of SOCS1 and SOCS3 action

### 2.1 SOCS1 and SOCS3 structures

SOCS1 and SOCS3 proteins are characterized by distinct functional domains contributing to their biological activities.

**Figure 1:**
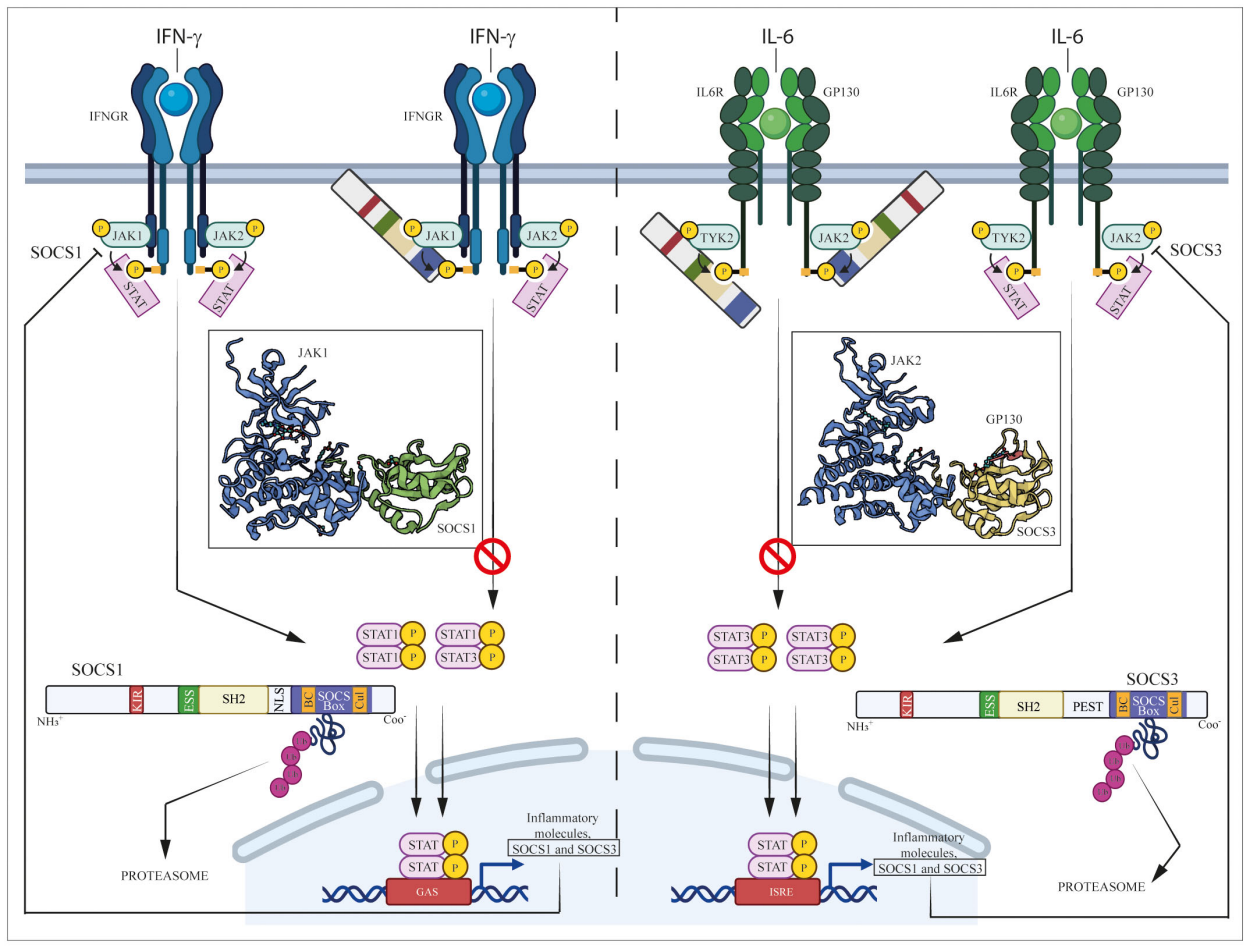
Regulation of JAK/STAT pathways induced by IFN-γ and IL-6 cytokine by SOCS1 and SOCS3. IFN-γ and IL-6 bind their specific IFN-γ and IL-6 receptors (IFNGR and IL-6R/GP130, respectively), causing the activation of associated intracellular JAK complexes (JAK1/JAK2 for IFN-γ and TYK2/JAK2 for IL-6 signaling), which mediate phosphorylation of tyrosine residues in components of receptor complexes. Docking site formation permit binding to receptors of STAT1 or STAT3, which form STAT dimers (STAT1/STAT1 or STAT1/STAT3 dimers for IFN-γ signaling, STAT3/STAT3 dimer for IL-6 signaling) following their phosphorylation. STAT homo- or heterodimers translocate into the nucleus where they activate expression of inflammatory molecules, as well as SOCS1 and SOCS3 themselves that provide negative feedback regulation.

Structurally, SOCS1 and SOCS3 share similarities in their overall architecture, both containing a SH2 domain flanked by a similarly sized N-terminal domain, a kinase inhibitory region (KIR) located upstream of the central SH2 domain and a 40-amino acid SOCS box domain in the C-terminal region (7–10) (**Figure 1**).

SH2 domain plays a pivotal role in interacting with phosphotyrosines present in target proteins, allowing SOCS1 and SOCS3 to bind to cytokine receptors and other proteins involved in signaling pathways (11). However, unlike the canonical SH2 domain found in many proteins, SH2 domains of SOCS proteins contain a unique N-terminal α-helical region, known as the extended SH2 (ESS) domain, that directly contacts residues crucial for phosphotyrosine binding (7). Immediately upstream of the ESS, both SOCS1 and SOCS3 contain KIR domain, a short amino acid stretch of 12 residues that acts as a pseudosubstrate, blocking the substrate binding groove of JAK to prevent detrimental kinase activity (12). Similar to SOCS1, SOCS3 also contains a C-terminal SOCS box domain able to assemble components of an E3 ubiquitin ligase complex through two motifs, the Elongin B/C (BC) box and the Cullin (Cul) box. This interaction with protein complexes mediates ubiquitination, leading to receptor degradation and thereby regulating the duration and intensity of signaling responses (13, 14). However, the main structural differences between SOCS1 and SOCS3 resides in the central region. Specifically, SOCS1 only contains a nuclear localization sequence (NLS) positioned between the SH2 domain and SOCS box, that mediates transport of SOCS1 into the cell nucleus and supports its role in terminating NF-kB signaling (15). On the other hand, SOCS3 features a 35-residue PEST motif between the SH2 domain and SOCS box that enhances SOCS3 turnover, influencing not only its degradation pathway but also its intracellular stability (7, 16). Both SOCS1 and SOCS3 play a key role in regulating immune responses, and their modular structure reflects the adaptability and specificity of cellular responses modulated by these proteins (**Figure 1**).

### 2.2 Inhibition of JAKs activity and related intracellular pathways by SOCS1 and SOCS3

The activation of the JAK-STAT pathway is integral to function of the largest group of immunomodulatory and inflammatory cytokines, including IL-2, IL-4, IL-13, IFN-α and IFN-γ. The activation of cytokine receptors results in the phosphorylation of intracellular receptor-associated JAK, which serves as docking sites for downstream transcription factors, such as members of the STAT family. Activated STAT dimers then translocate to the nucleus, where they bind to target elements, leading the transcriptional activation of multiple genes.

SOCS1 and SOCS3 are endogenous negative inhibitors of the activity of JAK and their related intracellular signaling pathways, which control the excessive and detrimental effects of cytokines trough different mechanisms. These mechanisms include the inhibition of JAK kinase activity, competitive binding with activated cytokine receptors and the targeting of proteins for degradation or rerouting (2, 17) (**Figure 1**). Aimed at inhibiting the tyrosine kinase activity, the N-terminal region containing KIR and the SH2 domains of SOCS3 is essential for impeding the catalytic activity of JAK1, JAK2, and TYK2. This inhibition is achieved through interaction with a GQM motif in JAK insertion loop. In particular, the first and third of these residues, G1071 and M1073, are indispensable for SOCS3 inhibitory function (18) (**Figure 1**).

The SH2 domain typically permits to SOCS proteins to directly interact with the exposed phosphotyrosines present on activated cytokine receptor. SOCS1 has been demonstrated to bind to a tyrosine residue (Y1007) on the JH2 domain of JAK2 proteins directly through its SH2 domain, leading to the inhibition of JAK2 catalytic activity (19). Furthermore, SOCS1 is able to bind JAK1, JAK3 and TYK2, inhibiting their activity (20). In contrast, SOCS3 binds JAK2 with a lower affinity and requires a higher expression than SOCS1 to achieve equivalent inhibition of kinase activity (21, 22).

Both SOCS1 and SOCS3 contain a KIR motif, essential for suppressing JAK tyrosine kinase activity. KIR domain acts as a pseudosubstrate blocking the substrate-binding groove of the JAK kinase domain and thereby prevents JAK from phosphorylating downstream substrates (13, 19). Babon et al. demonstrated that KIR binds to the surface of JH1 domain of JAK protein, rather than to the catalytic pocket, changing JH1 3D-conformation that inhibits phosphate transfer from ATP to the substrate peptide (23) (**Figure 1**).

SH2 domain present in both SOCS1 and SOCS3 is structurally similar to that of JAKs, and enables SOCS to compete with JAKs for binding to phosphotyrosines on cytokine receptors and sterically block binding of other molecules. Once bound to the activated receptor, SOCS1 and SOCS3 impede binding of other SH2 domain-containing proteins, such as STATs, thereby block STATs recruitment (17).

Both SH2 and KIR domains confer to SOCS proteins the specificity of inhibition of kinase activity or competitive binding with cytokine receptors. Indeed, although induced by many cytokines, each SOCS protein is specific for only a subset of cytokines. For example, SOCS1 is up-regulated by IL-2, IL-3, IL-6, IL-13, LIF, GM-CSF, IFN-γ, GH and prolactin but it regulates the intracellular signaling induced by the only IL-2, IL-6, IL-13, and IFN-γ cytokines (21, 24–30) (**Figure 1**).

SOCS1 appears to play an important role also in the regulation of other cytokines, such as IL-2, IL-4/13, IL-6, IL-12/23, IL-15 and the type-I IFNs (21, 31–36). Moreover, other than controlling the intracellular transduction of erythropoietin receptor by inhibiting JAK2/STAT5 signaling (37, 38), SOCS3 is involved in insulin and IGF-I signaling. Emmanuelli et al. (39) found that SOCS3 interacts directly with the insulin receptor on Tyr960, and inhibits insulin signaling by preventing STAT5β activation.

Considerable evidence showed SOCS3 exhibiting a high affinity for glycoprotein130 (gp130)-related receptors, especially IL-6 receptor (19, 40). The negative regulation of gp130 receptor subunit-mediated signaling by SOCS3 involves SH2 domain mediating interactions with a phosphorylated tyrosine residue (pY757) (41). These interactions induces a conformational change of SOCS3 that can bind to the surface of the JH1 kinase domain, including the GQM motif (42). Prolonged activation of STAT1 and STAT3 and induction of IFN-γ-inducible genes in response to IL-6 has been described in macrophages lacking SOCS3, suggesting an important role for preventing an IFN-γ-like response to IL-6 signaling (43).

Furthermore, SOCS3 is a leptin-inducible inhibitor of leptin signaling through specific bind to pTyr985 of leptin receptor. Leptin, secreted by adipocytes, activates the JAK/STAT pathway by binding to the long-form leptin receptor and SOCS3 represents a potential mechanism for leptin-resistant obesity (44, 45).

A third line of evidence shows that SOCS proteins act by promoting the degradation of specific signaling proteins. Indeed, Zhang et al. suggested that SOCS box-containing proteins act as adapter molecules that address activated signaling proteins to the proteasome machinery (46). Once bound to JAKs or receptors, SOCS box domain of SOCS1 and SOCS3 are able to interacts whit Elongin BC–Cullin5–RING-finger-domain that function as E3 ubiquitin ligases. E3 ligases define substrate specificity and covalently attach ubiquitin to lysine side chains in the substrate. Specifically, since SOCS proteins contain SH2 domains that bind tyrosine-phosphorylated signaling proteins, they can thus act as adapters that bring the E3 ubiquitin ligase complex in proximity of activated proteins, leading to their proteasomal degradation. SOCS proteins could themselves be ubiquitinated and degraded in this process (47) (**Figure 1**).

In conclusion, both SOCS1 and SOCS3 share a general strategy for JAK inhibition involving receptor interaction, competition for phosphotyrosines, blockade of catalytic activity, and ubiquitination followed by proteasomal degradation. However, they also exhibit specificity towards different JAKs and signaling pathways.

### 2.3 Regulation of SOCS1 and SOCS3 expression

SOCS mRNAs are triggered by cytokines, leading to the activation of SOCS proteins, which in turn inhibit cytokine-induced signaling pathways via a conventional negative feedback loop that influence its gene expression. The expression of SOCS1 and SOCS3 is not only influenced by several cytokines but also by hormones and growth factors (21, 48–50). Indeed, it has been found that that SOCS1 and SOCS3 are induced in fibroblasts by GH (51), whereas SOCS1 is up-regulated by insulin in liver and adipose tissue. SOCS1, in turn, negatively regulates insulin signaling by inhibiting insulin receptor substrate (IRS) proteins (52). Instead, leptin induces SOCS3 expression in keratinocytes, resulting in the inhibition of leptin signaling through negative feedback and insulin resistance, which interfered with their differentiation (53). However, the downstream transcriptional factors regulating SOCS1 and SOCS3 induction by growth factors and hormones remain unexplored.

This regulatory process occurs at both transcriptional and post-transcriptional levels, thereby contributing to maintaining a balance in immune and inflammatory responses (54, 55).

At the transcriptional level, SOCS1 and SOCS3 are regulated by transcription factors that can act as activators or repressors of transcription. STATs and NF-kB, for instance, can bind to regulatory elements within the promoters of the *socs1* and *socs3* genes, strongly inducing their expression (56–58). In particular, the transcriptional induction of SOCS1 by IFN-γ is mediated by STAT1 through IRF-1 binding to multiple GAAA sequences in the promoter region, rather than the gamma activated sequence (GAS) binding site typically used by the STAT1 homodimer (59). However, binding sites for STAT3 and STAT6 have also been identified within *SOCS1* promoter (56).

The *SOCS1* promoter contains putative STAT1-, STAT3- and STAT6-binding sites, with evidence suggesting that a dominant negative form of STAT3 can inhibit the IL-6-induced expression of SOCS1 mRNA (60). A study demonstrated that STAT1 acts indirectly on *socs1* transcriptional regulation by inducing the expression of IRF-1 transcription factor, which in turn stimulates transcription of the *SOCS1*gene (60). Moreover, it was found that Sp1 and IFN regulatory factor-1 transcription factors are responsible for the basal and IFN-γ–induced activity of socs1 promoter, respectively. The activity of the *SOCS1* promoter dependent on IFN-γ is also negatively regulated by two transcriptional repressors, namely, growth factor independence-1b (GFI-1b) and Kruppel-like factor 4 (KLF4), which tightly control SOCS1 transcription (61). Also SOCS3 expression is regulated by STATs. In fact, *SOCS3* promoter contains a STAT1/STAT3 binding element which is necessary and sufficient for its activation by LIF (62).

Of note, *SOCS* genes can be epigenetically silenced through the methylation of CpG islands or histone deacetylation (63, 64). *SOCS1* and *SOCS3* genes are often silenced by hypermethylation in several solid tumors, suggesting that hypomethylation plays a crucial role in disease onset and progression (65–67). For instance, epigenetic silencing of SOCS3 has been observed in malignant human melanoma. The repression of SOCS3 in malignant melanoma cells contributes to anti-apoptotic mechanisms and accelerates cell proliferation, suggesting that constitutive SOCS3 expression confers a proliferative advantage to human melanoma cells (68).

SOCS1 and SOCS3 mRNA can be subjected to post-transcriptional regulation through processes that can influence mRNA stability, subcellular localization, and translation of SOCS3 mRNA.

Specific elements within the 3’ UTR of SOCS3 mRNA can influence its stability. RNA interference or mRNA-binding proteins can regulate its degradation or stabilization.

Both genes can be subjected to regulation via RNA interference mechanisms, including the action of microRNAs that typically bind to sites located in the 3′UTR. This interference can disrupt the translation process or promote mRNA degradation (69–71).

Evidence indicated that miR-155 drives Treg/Th17 cells differentiation and enhances Th17 cell function by directly inhibiting SOCS1 protein expression (72). Interestingly, several studies showed that miR-155 promotes inflammatory cytokine expression and IFNs response in primary macrophages and dendritic cells (73).

Based on bioinformatic analysis, SOCS1 was identified as a putative target gene for miR-19b-3p and was also a negative regulator of NF-κB signaling pathway. *In vitro* studies showed that both mRNA and protein expressions of SOCS1 were inhibited and miR-19b-3p was internalized by macrophages, leading to M1 phenotype polarization (74).

With the aim of discovering new treatment targets, it was observed that miR-30a negatively regulated SOCS1. High miR-30a levels and reduced SOCS1 expression led to increased levels of JAK2, STAT3, Bax, TLR4, and HMGB1 as well as to the extent of JAK2 and STAT3 phosphorylation. By targeting and negatively regulating SOCS1 via the JAK/STAT signaling pathway, miR-30a inhibited the liver cell proliferation and promoted cell apoptosis in rats with sepsis (75).

Furthermore, miR-122 is involved in enhancing IFN-α signaling in the liver by regulating *SOCS3* promoter methylation (76). miR-19a can also control SOCS3 expression by binding the 3’-UTR and functionally contributes to the regulation of anti-viral and pro-inflammatory responses induced by IFNs (70).

According to Sonkoly et al., the downregulation of SOCS3 in psoriatic lesions is associated with the upregulation of miR-203 (77). Xu et al. identified an opposite link between miR-203 and SOCS3 expression in IL-17-activated HaCaT cells (78). Taken together, these findings suggest that SOCS3 is a direct target of miR-203 and may influence the IL-17-induced VEGF production. The suppression of SOCS3 by miR-203 adds complexity to SOCS3 regulation, potentially impacting keratinocyte functions in both developing and adult skin (79).

Post-translational modifications, such as phosphorylation of the SOCS box, can regulate the activity and stability of SOCS proteins, influencing their ability to suppress signaling pathways (80, 81). Finally, SOCS3 features a PEST motif adjacent to its SH2 domain, providing an additional mechanism to influence protein stability (7).

## 3 Regulation by SOCS1 and SOCS3 of skin inflammation and malignant transformation

The skin serves as a crucial barrier between the body and the external environment, and is essential for homeostasis maintaining a delicate balance in immune responses. The control of cell proliferation is also fundamental in the skin context, as aberrations in cell-growth regulatory mechanisms contribute to skin tumorigenesis.

STAT proteins play a pivotal role in mediating intracellular responses to cytokines, and SOCS, in particular SOCS1 and SOCS3, finely regulate these signaling cascades by preventing excessive or prolonged proinflammatory and pro-proliferative responses. Aberrant STAT hyperactivation and consequent SOCS1 or SOCS3 intervention is observed in immune-mediated inflammatory diseases and malignancies of the skin.

### 3.1 Definition of SOCS1 and SOCS3 roles in psoriasis and atopic dermatitis

SOCS1 and SOCS3 are expressed in various cell types within the skin, including keratinocytes, fibroblasts, and immune cells. During development of skin inflammatory diseases, keratinocytes, the predominant cell type in the epidermis, express both SOCS1 and SOCS3 molecules in response to a plethora of IFNs and ILs, mostly derived from T lymphocytes, which inhibit in a negative feedback loop the JAK/STAT pathways activated by these inducing stimuli (82). Among them, type-1 (IFN-γ), type-2 (IL-4, IL-13) and IL-10 superfamily (IL-10, IL-20, IL-22) cytokines are potent inducers of SOCS1 and SOCS3 in keratinocytes (82–86), with different consequent effects.

Concerning IFN-γ, it aberrantly influences the inflammation and apoptotic/growth rate in keratinocytes, and in parallel induces SOCS1 and SOCS3 which in turn downregulate the expression of inflammatory genes, such as major histocompatibility complex class (MHC)-II, ICAM-1, and CXCL10, CXCL9, and CCL2 chemokines (82). Moreover, keratinocytes overexpressing SOCS1 show low susceptibility to IFN-γ-mediated growth inhibition, and constitutively show higher levels of the mitogenic cytokine CXCL8, and of RAS/ERK1/2 activities (82, 87).

Interestingly, keratinocytes of psoriasis skin express increased levels of SOCS1, as compared to healthy cells, as consequence of an unbalanced binding of transcriptional activators and repressors to SOCS1 promoter after IFN-γ stimulation (61). In fact, while Sp1 and IRF-1 transcription activators of SOCS1 are expressed at similar levels in keratinocytes of healthy and psoriasis donors, the transcriptional repressors of SOCS1, the GFI-1b and KLF4, are downregulated in psoriatic cells. SOCS1 upregulation could represent a mechanism in psoriatic keratinocytes through which these cells attempt to protect themselves from IFN-γ-induced inflammatory effects (88).

Similarly to SOCS1, SOCS3 is also upregulated in psoriasis epidermis, especially in areas close to CD3^+^ dermal infiltrate likely producing inflammatory cytokines (89). IFN-γ/TNF-α-treated keratinocytes of psoriasis patients strongly express SOCS3 and SOCS1, at higher levels as compared to healthy cells. In psoriasis, SOCS3 and SOCS1 suppress cytokine-induced apoptosis by sustaining the activation of the PI3K/AKT pathway in keratinocytes. The latter determines the activation of the anti-apoptotic NF-κB cascade and, in parallel, the inhibition of the pro-apoptotic BAD function in keratinocytes (89). Both AKT and BAD are strongly expressed in lesional skin and contribute to the increase of dysfunctional apoptosis and, thus, the peculiar thickening of psoriatic skin (90). In addition, SOCS3 has been shown to be involved in controlling keratinocyte proliferation, as demonstrated in transgenic mice overexpressing SOCS3 in keratinocytes (tsgn-K5/SOCS3) of the basal layer of epidermis (86). Tsgn-K5/SOCS3 mice show full inhibition of STAT3 phosphorylation and atrophied epithelia as result of a marked reduction of proliferating cells and total keratinocyte numbers. Therefore, experimentally mimicked SOCS3 overexpression might turn out to be beneficial in psoriasis by reducing STAT3-dependent inflammation and proliferation. In contrast, SOCS3 overexpression could be deleterious in a hampered cutaneous wound healing context, such as diabetic ulcer, where keratinocyte proliferation and inflammatory responses are fundamental to restore skin integrity after damage. The crucial role of SOCS3 in maintaining homeostasis and preventing skin inflammation has also been demonstrated in SOCS3 gene deficient mice (86). In fact, *Socs3* cKO causes severe skin inflammation with epidermal hyperplasia and enhanced inflammatory gene expression. The inflamed skin showed constitutive STAT3 activation and up-regulation of IL-6 and IL-20 receptor (IL-20R) related cytokines (i.e. IL-19, IL-20 and IL-24). Disease symptoms can be counteracted by deletion of *Il6* gene, but not by the deletion of *Il23, Il4r*, or *Rag1* genes. The expression of IL-6 in *socs3* cKO keratinocytes increases expression of IL-20R-related cytokines that further facilitates STAT3 hyperactivation, epidermal hyperplasia and neutrophilia. These results demonstrate that the IL-6-STAT3-SOCS3 axis strictly regulates skin homeostasis. Moreover, the SOCS3-mediated negative feedback loop in keratinocytes has a critical mechanistic role in the prevention of skin inflammation caused by hyperactivation of STAT3 (86).

Under the influence of proinflammatory cytokines released by leukocytes infiltrating the dermis, also psoriatic dermal fibroblasts upregulate SOCS3 (91). This increased SOCS3 expression correlates with the enhanced activation of TGF-β signaling pathway in psoriasis microenvironment, and elevated production of extracellular matrix (ECM) components, such as collagen and fibronectin, thus indirectly impacting inflammation and excessive proliferation of keratinocytes (92). Indeed, TGF-β can deregulate SOCS3 expression in fibroblasts by inducing the hypermethylation of its promoter and mRNA silencing. As a consequence of SOCS3 silencing, STAT3 is hyperactivated and fibroblasts highly release collagen and promote fibrosis *in vitro* and *in vivo* (92). Furthermore, SOCS3 can be responsible for fibroblast proliferation and migration by activating p120 Ras-Gap and ERK1/2 signaling in response to IL-6 and other mitogenic cytokines released in psoriatic skin (93, 94).

Central to psoriasis pathogenesis is the dysregulated signaling of cytokines influencing pathogenic immune responses, particularly IL-23 and IL-17A (88). The pro-inflammatory cytokine IL-23 is critical for the differentiation and maintenance of Th17 cells, and SOCS1 greatly influences IL-23-dependent STAT3 signaling downstream of IL-23 receptor in Th17 cells (94). In psoriasis, the diminished expression of SOCS1 in T cells would contribute to the sustained activation of IL-23-driven pathways and differentiation of Th17 lymphocytes, thus fostering an environment conducive to chronic inflammation and aberrant keratinocyte proliferation. In parallel, dysregulation of SOCS1 in psoriasis has been associated with enhanced dendritic cell activation, resulting in an increase of autoreactive T-cell priming s and perpetuation of the immune cascades (95).

AD, characterized by pruritic and inflamed skin lesions, represents another immune-mediated skin disease where SOCS1 and SOCS3 involvement is evident. The pathogenesis of AD involves a complex interplay of immune dysregulation, barrier dysfunction, and a predisposition to allergic reactions (96).

IL-4 and IL-13, cytokines associated with allergic inflammation, play a pivotal role in AD pathogenesis. SOCS1 and SOCS3, as negative regulators of JAK1/JAK3/STAT6 and JAK1/TYK2/STAT3/STAT6 signaling downstream of IL-4 and IL-13 receptors (97), influences the intensity and duration of these inflammatory responses. SOCS3 has been shown to have an important role in regulating the onset and maintenance of Th2-mediated allergic and atopic diseases, such as AD and asthma (98). In fact, SOCS3 transgenic mice overexpressing SOCS3 in T cells show increased Th2 responses and multiple pathological features typical of asthma in an airway hypersensitivity model system. In contrast, Th2 responses are deficient in both homozygous and heterozygous SOCS3 −/− knockout transgenic mice (98). Consistently, SOCS3 in Th2 cells evokes type-2 cytokine production, IgE production and eosinophilia by inhibiting Th1 differentiation process and thus controlling the balance between Th1 and Th2 cells (99). On the other hand, SOCS3 and SOCS1 in resident AD fibroblasts can impact the recruitment of Th2 cells and eosinophils by deregulating the IL-4-induced expression of the chemokine eotaxin, achieved through the direct inhibition of STAT6 (91).

Beyond its role in cytokine signaling, SOCS3 can influence the maintenance of the epidermal barrier. AD is characterized by immune cell infiltrates and impaired barrier function. SOCS3, by modulating IL-4 and IL-13-dependent signaling pathways regulating the expression of filaggrin, may indirectly impact the progression and severity of AD. Finally, AD is also complicated by recurrent infections, which have been associated with decreased or impaired production of the antimicrobial peptide defensins. Among them, the β-defensins HBD-2 and HBD-3 are downregulated in AD, as consequence of STAT1 inhibition by SOCS1 and SOCS3, transcriptionally activated by IL-4 and IL-13 via STAT6 (83). Due to their role in modulating fibrosis and fibroblast collagen deposition, SOCS1 and SOCS3 may regulate the aberrant responses and excessive ECM production induced by IL-4 and IL-13 in fibroblasts, leading to dermal fibrosis and impaired wound healing characterizing AD skin (92).

SOCS1 and SOCS3 control on immune-mediated skin diseases also depends on the effects that they exert on T-cell polarization. SOCS1 has been shown to downregulate Th1 differentiation by inhibiting IFN-γ-mediated STAT1 activation (100). Accordingly, deficiency of SOCS1 in CD4^+^ T cells determines increased Th1 responses due to the upregulated IFN-γ signaling, but also indirectly through reduced IL-6/STAT3-mediated Th1 suppression mediated by SOCS3 (101). SOCS1 can also inhibit IL-4-mediated STAT6 activation that drives Th2 polarization, and IL-12-mediated Th1 polarization (102). SOCS1−/− IFN-γ−/− knockout mice were skewed toward Th2 responses, unveiling the role of SOCS1 in the inhibition of Th2 polarization (103). In addition, loss of SOCS1 in CD4^+^ T cells suppresses Th17 cell development (101).

Genetic factors play a significant role in predisposing individuals to psoriasis or AD. Research has identified specific genetic variations, or polymorphisms, in both *socs1* and *socs3* genes that may predispose to psoriasis and atopy conditions. Among them, rs4780355 and rs33989964 single-nucleotide polymorphism (SNP) in SOCS1 associate with psoriasis and asthma conditions, respectively (104, 105). On the other hand, rs8074003 SNP in SOCS3 predisposes to atopic disease development, maybe leading to increased SOCS3 expression and IL-4 levels in AD patients (106). These SNPs could influence the expression levels of SOCS and/or their function on JAK/STAT signaling and specific signaling molecules. As a consequence of genetic variations presence in psoriasis or AD patients, the fine-tuning of immune responses could be compromised and potentially tip the balance towards aberrant inflammation.

### 3.2 Regulation by SOCS1 and SOCS3 of malignant skin transformation

SOCS1 and SOCS3 proteins were initially described as onco-suppressors, being silenced in many tumors (42) as consequence of hypermethylation of their promoters or mutations influencing proliferation, differentiation and survival of immune cells controlling tumor expansion (107). SOCS1 and SOCS3 expression and anti-tumor function have been also elucidated in skin cancer contexts, such as non-melanoma skin cancer (NMSC) and melanoma.

NMSC constitutes a prevalent group of malignancies, with basal cell carcinoma (BCC) and squamous cell carcinoma (SCC) as its primary subtypes (108). These cancers arise from the neoplastic transformation of keratinocytes of the basal or suprabasal layers of epidermis, driven by cumulative sun exposure, genetic factors, and immune dysregulation. SOCS1 and SOCS3 have garnered increasing attention for their intricate involvement and multifaceted roles in the pathogenesis of NMSC. In BCC and SCC skin lesions, SOCS1 and SOCS3 are moderately detected *in vivo* (85). Their decrease commonly associates with a transcriptional silencing or selective unresponsiveness to the inducing cytokines. Consistently, hyper-methylation of the *SOCS3* promoter is found in 90% of head and neck cancer (109), suggesting that *SOCS3* inactivation induced by methylation gene may be an early event in these cancers. DNA hypermethylation of *SOCS1* is also frequently found in certain types of lymphomas, with a consequent enhancement of STAT1 and JAK2 activity and hence cell proliferation (110). SOCS1 and SOCS3 expression is reduced in tumor lesions of BCC and SCC, as compared to other skin inflammatory conditions such as psoriasis, despite the high number of IL-22-secreting tumor-infiltrating lymphocytes (85). The latter cells, together with IL-17-producing T cells, promote tumor progression in NMSC by upregulating proliferation and pro-survival pathways dependent on STAT3, NF-κB, and AKT signaling (111). Interestingly, IL-22 is not able to induce SOCS3 transcriptional expression in BCC-or SCC-derived keratinocytes, contrarily to healthy cells. SOCS3 deficiency in NMSC indirectly sustains tumorigenesis by enabling aberrant activation of IL-22-induced STAT3, which promotes cell-cycle progress and suppresses anti-apoptotic events. Potential target genes of STAT3 are involved in cell survival, including Bcl-2 and Bcl-xL, and cell cycle regulators, such as cyclin D1 and cyclin E1, and p21 (112). Vascular endothelial growth factor (VEGF), which is among the target of STAT3, also contributes to tumor angiogenesis (113). Of note, *in vitro* and *in vivo* studies conducted on SCC models showed that SOCS3 (and SOCS1) forced expression by SOCS3-derived peptide (KIR-ESS, see paragraph 4.1) efficiently suppresses IL-22-induced tumor growth (85).

SOCS1 and SOCS3 are key players also implicated in melanoma cell growth and tumor development, even if controversial effects have been described. In early studies, SOCS1 was considered as a progression marker of human melanoma, being its expression related to tumor invasion, tumor thickness and stage of the disease (114). Consistently, SOCS1 inhibition in murine B16F10 melanoma cells has been shown to revert the tumorigenic phenotype and inhibit cell metastasis, by inducing cell-cycle arrest at S-phase and decreasing melanoma cell invasion (115). In addition, down-regulation of SOCS1 was capable to decrease the activation of different pro-tumorigenic signals in melanoma cells (115). On the other hand, SOCS1 has been found to block mitosis in melanoma cell lines, by reducing levels of cell-cycle G1 phase regulators, such as cyclin D and cyclin E levels and altering M-phase protein levels (116). SOCS1 also significantly diminished the metastatic capability of melanoma cells, as consequence of its negative control of STAT3 signaling and matrix metalloproteinase-2, basic fibroblast growth factor, and VEGF expression (117).

Similarly to SOCS1, the role of SOCS3 in melanoma appears to be context-dependent, exhibiting both tumor-suppressive and pro-tumorigenic functions (118). Constitutive expression of SOCS3 has not reported for all melanoma cell lines, and high SOCS3 expression has been correlated with insensitivity to the growth-inhibitory effect of IL-6 (119), IFN-γ and IFN-α (120). Suppression of SOCS3 re-established the growth-inhibitory role of IL-6. Consistently, the targeted inhibition of SOCS3 activity in macrophages determined the suppression of melanoma metastasis and prolonged survival in mice xenografted with B16F10 cells (121). On the other hand, CCR5 blockade suppresses melanoma development through inhibition of IL-6-STAT pathway via upregulation of SOCS3 (122). Finally, evidence of a direct role of SOCS3 in tumor progression in terms of disruption of cell cycle does not exist, likely due to the fact that SOCS3, differently from SOCS1, cannot localize in the cell nucleus, since it lacks a nuclear translocation domain (15).

## 4 SOCS1 and SOCS3 as “first-generation” models for the development of therapeutic JAKs inhibitors

### 4.1 Design and functional characterization of KIR-derived peptides mimicking SOCS1 and SOCS3

The development of SOCS1 and SOCS3 mimetic peptides started from the detailed characterization of the activation/autophosphorylation loop of JAK2, with the aim at inhibiting its activity independent of the regulation of JAKs by SOCS (123). This study permitted to identifying the amino acid region LPQDKEYYKVKEP of human JAK2, depicted symbolically as pJAK2(1001–1013) (with the p of pJAK2 indicative of phosphorylation of tyrosine 1007), as a focal starting point for the design of SOCS1 mimetic peptides (124, 125).

A first 12-amino acid SOCS1 mimetic peptide, named Tkip, was developed by explorating hydropathic complementarity to the JAK2 activation loop and was characterized by the sequence WLVFFVIFYFFR (126, 127). This predicted peptide showed homology with the F56, F59, and R60 residues of KIR sequence and bound the activation loop of JAK2, thus inhibiting IFN-γ-induced activation of JAK2 and STAT1α, as well as phosphorylation of IFN-γ receptor subunit IFNGR1 (128). Functionally, Tkip inhibited antiviral activity of IFN-γ, as well as expressipon of MHC class I molecules in fibroblasts (128, 129). Furthermore, Tkip reduced inflammatory symptoms in murine encephalomyelitis model, by inhibiting IFN-γ signaling and suppressing the effector functions of T-cells (130, 131) (**Table 1**).

**Table 1:** List of SOCS1 and SOCS3 mimetic peptides used in preclinical studies.

PEPTIDE	SEQUENCE	FUNCTION	*IN VITRO* AND *IN VIVO* EMPLOYMENT	REFs
Tkip	WLVFFVIFYFFR	SOCS1 mimetic	Murine model of lethal poxivirus infection; murine encephalomyelitis model	(126–131)
pJAK2 (1001–1013)	^1001^-LPQDKEpYYKVKEP	SOCS1 antagonist	*In vitro* assay of SARS-CoV-2 replication	(127)
SOCS1-KIR	^53^-DTHFRTFRSHSDYRRI	SOCS1 mimetic	Ex-vivo models of skin inflammation; rat experimental model of autoimmune uveitis	(84, 127, 132)
R9-SOCS1-KIR	RRRRRRRRRDTHFRTFRSHSDYRRI	SOCS1 mimetic	Endotoxin-induced uveitis	(133)
new KIR	^53^-DTHFRTFRSH	SOCS1 mimetic	*In vitro* binding assays	(134)
PS-5	DTC(Acm)RQTFRSH	SOCS1 mimetic	*In vitro* and ex-vivo models of skin inflammation; *in vitro* and *in vivo* atherosclerosis mouse model	(84, 134–137)
KIR-SOCS3	^22^-LKTFSSKSEYQL	SOCS3 mimetic	*In vitro* binding assays; *in vitro* assays on IL-22-stimulated keratinocytes	(138, 139)
ESS-SOCS3	^34^-VVNAVRKLQESG	SOCS3 mimetic	*In vitro* binding assays	(138, 139)
KIR-ESS-SOCS3	^22^-LKTFSSKSEYQLVVNAVRKLQESG	SOCS3 mimetic	*In vitro* assays on IL-22-stimulated keratinocytes; mice bearing squamous cell carcinoma xenografts; mouse xenografts of triple negative breast cancer; *in vitro* model of atherogenis	(85, 139, 140)
KIRCONG-chim	^25^-FSSKSEYQLβAlaβAlaFYWSAVT	SOCS3 mimetic	*In vitro* model of atherogenis	(139)

As previously described, SOCS1 and SOCS3 KIR region of are located in the N-terminus of the proteins and are adjacent to a similarly short ESS sequence. Peptide corresponding to the ESS sequence was not able to bind the pJAK2(1001–1013) peptide. Thus, Tkip fortuitously led to the KIR region of SOCS1 as a potential SOCS1 mimetics. Analysis of dose–response competitive binding suggested that Tkip and SOCS1-KIR recognized the activation loop of the pJAK2(1001–1013) peptide with similar affinity (127). Differently from Tkip, SOCS1-KIR did not inhibit JAK2 autophosphorylation. However, both peptides inhibited STAT1α activation, IFN-γ-induced activation of macrophages, and antigen-specific lymphocyte proliferation. Of note, topical administration of SOCS1-KIR peptide successfully prevented uveitis and ocular damage (132). Furthermore, a modified version of SOCS1-KIR, named R9-SOCS1-KIR, containing a N-terminal poly-arginine sequence for penetration into plasma membrane, ameliorated autoimmune uveitis in mice (133) (**Table 1**).

In 2012, a new mimetic peptide of the kinase-inhibitory region of SOCS1, named PS-5, was designed (134). In detail, staring from the 52–67 KIR sequence of SOCS1, through an alanine scanning approach, a truncated linear peptide (62–61), named new KIR, was designed, showing ability to bind to JAK2 (**Table 1**). Subsequently, new KIR was transformed in a new lead compound, named PS5, carrying the mutations His54/Cys(Acm), Phe55/Arg, and Arg56/Gln, by using a screening of “combinatorial focused libraries” in positional scanning (PS) format (141–143). The new unnatural sequence DTC(Acm)RQTFRSH of PS5 improved JAK2 binding by establishing enhanced electrostatic interactions with the negative phosphate moiety on Y1007 of JAK2. In addition, due to the presence of non-natural residue (Cys (Acm), PS-5 exhibited greater protease stability and effectively inhibited STAT1 phosphorylation and expression levels of IRF-1 expression in human keratinocyte cultures (134). Ps-5 peptide showed also antioxidant and athero-protective effects in *in vitro* and *in vivo* model of atherosclerosis (144) (**Table 1**). Un-natural residues and a lactam internal bridge were further introduced within SOCS1-KIR motif of PS-5 mimetic, generating a set of internal cyclic PS5 analogues These new peptides were able to reduce cytokine-induced proinflammatory gene expression, oxidative stress generation and cell migration, by inhibiting JAK-mediated tyrosine phosphorylation of STAT1 and to (135). Starting from the cyclic peptidomimetic of KIR-SOCS1, icPS5(Nal1), identified in the previous study, La Manna et al. designed novel derivatives, containing crucial amino acids substitutions and/or modifications affecting the ring size that ensured the maintenance of low-micromolar affinity toward JAK2, and a great serum stability (136).

In 2013, the crystal structure of the ternary complex among murine SOCS3/JAK2 kinase domain/gp130 phosphotyrosine-peptide was resolved and allowed to design mimetics of SOCS3 (145). From its inspection, Leu22, Lys23, and Thr24 residues of KIR domain and Val34, Val35, and Val38 of ESS domain of SOCS3 appeared to interact with the GQM” and helix G motifs of JAK2 through hydrophobic interactions (40). Additional contacts involved a “hinge” region between ESS and helix A (HA) of SH2, where the stretch 46–52, called CONG, bears three adjacent aromatic residues, FYW. With the aim to obtain SOCS3 proteomimetics, the ability of different linear peptides, overlapping KIR domain, was analyzed in their ability to recognize JAK2 and mimic SOCS3 cellular effects. Initially, KIR-SOCS3 and ESS-SOCS3 peptides were analyzed isolated and in combination: the entire KIR-ESS-SOCS3 region exhibited good affinity for JAK2 and an efficient suppression of both IL-22 signaling in keratinocytes (85). Aimed at exploring the contribution of other SOCS3 regions, the CONG region was transformed in a chimeric peptide, named KIRCONG chim, that contained non-contiguous fragments: a reduced region of KIR (25–33) and CONG (46–52) connected by β-alanines as spacers (144). This proteomimetic showed anti-inflammatory properties in vascular smooth muscle cells and macrophages, by suppressing STAT3 phosphorylation, as well as the expression of genes as *CXCL10* and *CCL5* chemokines and the superoxide-generating enzyme *NOX2* (138, 139). The observed anti-inflammatory and antioxidant properties of KIRCONG chim corroborated the potential application of SOCS3 mimetics in inflammatory diseases. However, their direct use as drugs are hampered by low aqueous solubility and high molecular weights (**Table 1**).

### 4.2 Effects of SOCS1 and SOCS3 manipulation in preclinical model of psoriasis and non-melanoma skin cancer

Pathogenic mechanisms leading to the manifestation of immune-mediated skin disorders, such as psoriasis and allergic contact dermatitis, are mostly driven by Th1 and Th17 lymphocytes (146, 147). Other than IFN-γ and IL-17, Th1 and Th17 cells can release high amounts of TNF-α, which act in synergy with IFN-γ and IL-17 in reinforcing the inflammatory responses of target cells, among which epidermal keratinocytes (148). Th22 cells also contribute to disease pathogenesis, stimulating defined signaling pathways in epidermal keratinocytes, in several immune-mediated skin diseases (146). Despite recent studies demonstrating that IL-23/IL-17 axis has a pathogenic role in the development of psoriasis, IFN-γ remains a crucial cytokine trigger of resident skin cells, as it potently enhances the expression of pro-inflammatory genes in epidermal keratinocytes activated by the transcription factor STAT1 and alters their apoptotic/growth rate. These pro-inflammatory genes include IRF-1 transcription factor, as well as chemokines and adhesion molecules that contribute to recruitment of leukocytes into the inflammatory sites (149, 150).

**Figure 2:**
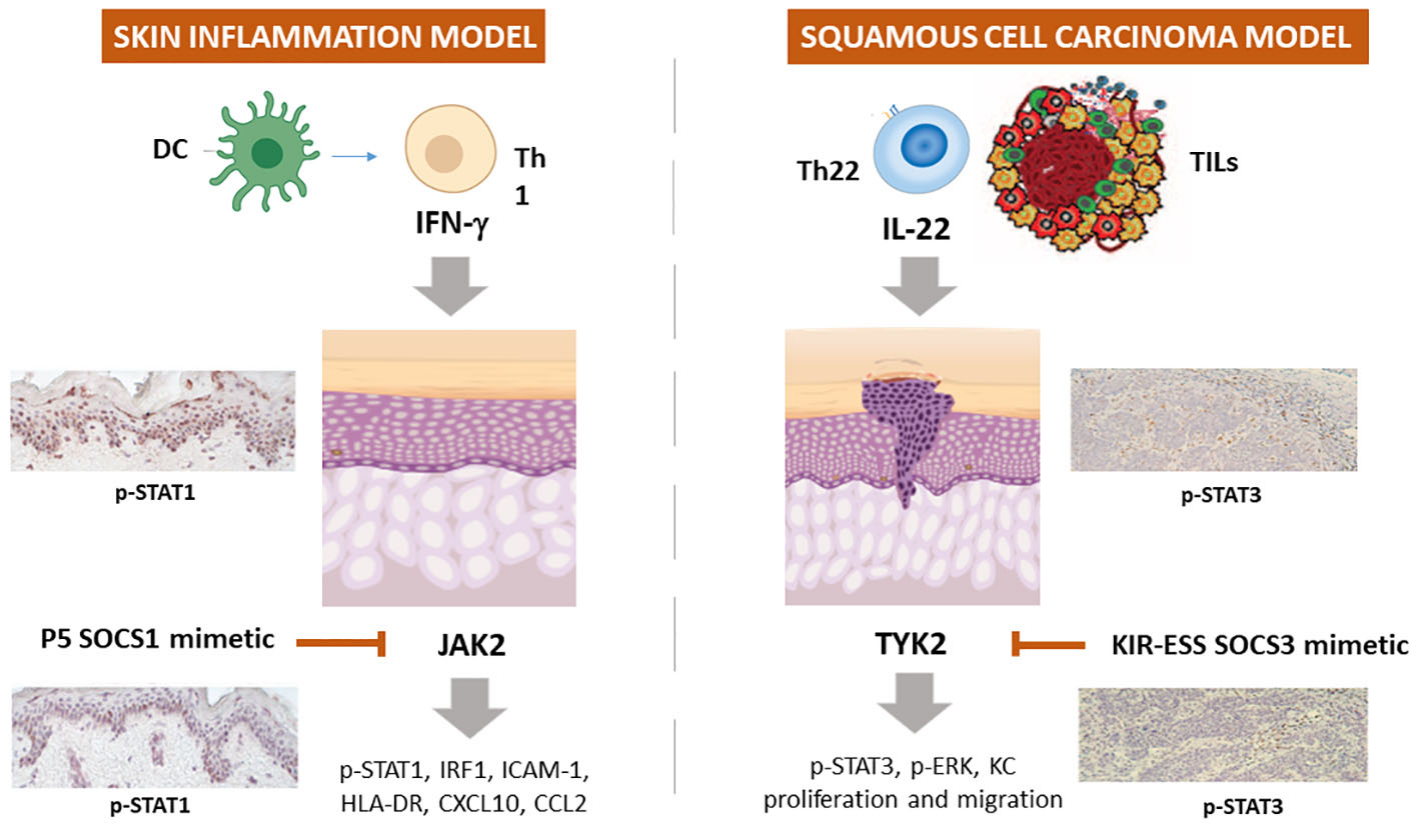
Schematic representation of the employment of SOCS1 and SOCS3 mimetic peptides in experimental models of skin inflammation and non-melanoma skin cancer. In IFN-γ-activated keratinocytes or skin explants, SOCS1 and its mimetic peptide PS-5 inhibit the expression of ICAM-1, HLA-DR, CXCL10, and CCL2 through the blockade of JAK2 tyrosine kinase activity, that leads to STAT1 and IRF-1 activation. In IL-22 activated keratinocytes and skin biopsies of squamous cell carcinoma murine model, KIR-ESS SOCS3 peptide inhibits the expression of p-STAT3 and p-ERK1/2, as well as keratinocyte proliferation and migration.

We definitively assessed that SOCS1 negatively regulates the molecular cascades triggered by IFN-γ by disabling JAK2 phosphorylation in epidermal keratinocytes (82, 87). Based on this knowledge, the therapeutic potential of PS-5 has been explored in managing pathogenesis of inflammatory skin diseases mediated by IFN-γ (84, 137). In particular, the ability of the PS-5 SOCS1 mimetic peptide, compared to that of SOCS1-KIR peptide, in suppressing the inflammatory responses was evaluated in IFN-γ-activated epidermal keratinocytes by using *in vitro* cultures and whole-skin explants exposed to IFN-γ. We found that PS-5 efficiently suppressed the IFN-γ molecular signaling in keratinocytes, for instance the JAK2-STAT1-IRF-1 cascade, as well as the downstream expression of STAT1-IRF-1-dependent genes (84, 137). As a direct consequence of the inhibition of CXCL10 and CCL2 chemokine release and partial reduction of ICAM-1 induction, PS-5-treated keratinocytes could no longer retain and induce migration of T lymphocytes in response to IFN-γ. In addition, human skin explants treated with PS-5 did not exhibit the inflammatory signature typically induced by IFN-γ, confirming the ability of this peptide in reducing the inflammatory responses of IFN-γ-activated keratinocytes (84, 137) (**Table 1**; **Figure 2**).

Epidermal keratinocytes are also involved in BCC and SCC and IL-22-releasing TILs participates to BCC and SCC growth by inducing keratinocyte proliferation and migration, as well as the expression of inflammatory, anti-apoptotic and pro-angiogenic genes. In 2017, we performed a study aimed at rescuing the decreased SOCS3 activity in these tumor contexts by using a SOCS3-derived KIR-ESS peptide (85). We found that SOCS3-KIR-ESS peptide suppressed the IL-22 molecular signaling in keratinocytes, by acting on IL-22R1 and JAK1 phosphorylation and STAT3 and Erk1/2 activation, as well as on the expression of STAT3-dependent downstream genes. As a direct consequence, KIR-ESS peptide counteracted *in vitro* proliferative and migratory potential of NMSC-derived keratinocytes induced by IL-22. Consistently with *in vitro* results, KIR-ESS peptide reduced tumor growth in athymic nude mice bearing SCC xenografts, especially when xenografts were subjected to IL-22 administration (85). Inhibition of tumor growth by KIR-ESS peptide was associated to its efficacy in reducing phosphorylation of STAT3 within tumor where keratinocytes are responsible for maintaining tumor growth (151). In line, KIR-ESS peptide showed a significant inhibition of primary tumor growth and pulmonary metastasis in mouse xenografts of MDA-MB-231-luci tumors usually used as models of human triple negative breast cancer (140).

As a whole, these data provided a rationale for the use of peptides mimicking the action of SOCS1 and SOCS3 in the amelioration of skin inflammation, as well as of skin BCC and SCC tumors (**Table 1**; **Figure 2**).

### 4.3 Development of small molecules inhibiting JAKs activity (JAKs inhibitors)

Despite various strategies are currently being employed to improve peptide properties, mimetic peptide drugs present limited permeability, proteolytic vulnerability, short half-life, swift *in vivo* clearance, and limited oral bioavailability (152). However, the design of peptides mimicking the JAKs inhibitory region of SOCS1 and SOCS3 proteins and their employment in managing pathogenesis of inflammatory immune-mediated skin disease led the way towards the development of a class of small molecules inhibiting specific JAKs, known as JAK inhibitors. JAK inhibitors are designed to modulate aberrant JAK activity and downstream signaling pathways, providing a targeted approach for treating diseases driven by dysregulated immune responses. Similarly to SOCS1 and SOCS3 KIR regions, these inhibitors selectively target JAK enzyme family, which plays a crucial role in the JAK/STAT signaling pathway involved in immune response and inflammation. In detail, JAK inhibitors exhibit anti-inflammatory effects suppressing cytokine production involved in Th1, Th2, Th17, and Th22 immune pathways (153). This mechanism contrasts with that of biological disease modifying drugs, which are monoclonal antibodies targeted against only one or two specific cytokines, such as TNF-α, IL-23 or IL-17 blockers for psoriasis or IL-4 and IL-13 inhibitors for atopic dermatitis.

**Figure 3:**
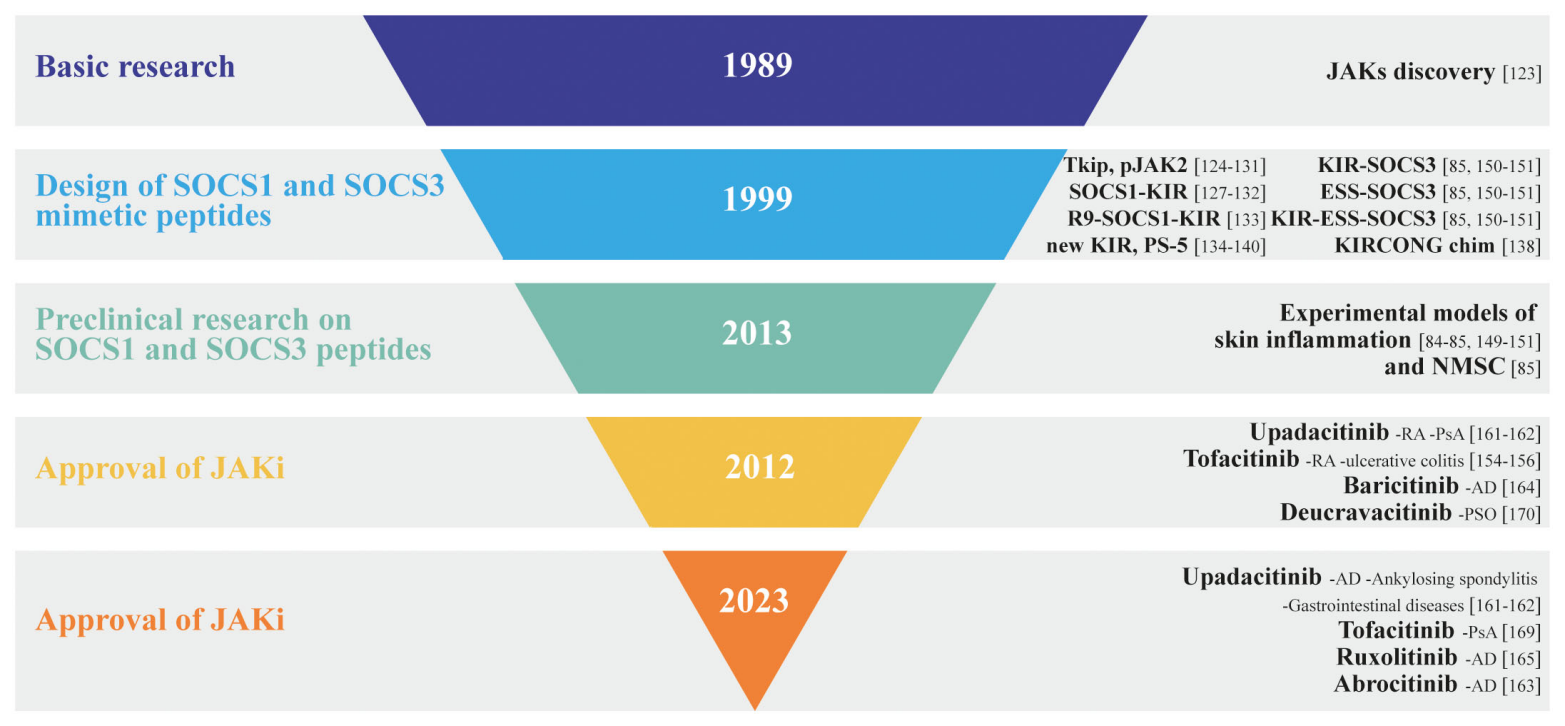
Inverted pyramid model illustrating the design and employment of SOCS1 and SOCS3 mimetic peptides in skin disorders, as well as the discovery and approval of JAK inhibitors, highlighting the key milestones and stages in the process.

The development of JAK inhibitors has undergone several phases, from early preclinical research to extensive clinical trials. To date, several JAK inhibitors have received the US Food and Drug Administration (FDA) approval for specific autoimmune/inflammatory disorders and are currently being evaluated for additional conditions. Depending on selectivity, JAK inhibitors can be classified as first-generation or poor-selective (e.g., tofacitinib, baricitinib, ruxolitinib) or second generation (e.g., upadacitinib, abrocitinib) inhibitors, with the latter being considered the most selective for each JAK subtypes (**Figure 3**).

Tofacitinib was the first JAK inhibitor approved by the FDA for treating moderate-to-severe rheumatoid arthritis, psoriatic arthritis (PsA), and ulcerative colitis in adult patients. Being a first-generation JAK inhibitor, it is non-selective by acting against JAK1 and JAK3 isoforms along with some JAK2 inhibition. Since all JAK isoforms are inhibited, it effectively blocks several gamma chain cytokines including IL-2, IL-4, IL-15 and IL-21 (154). Tofacitinib is capable of reducing TNF-α, IL-1β, and type I IFN production in dendritic cells stimulated with antigenic lipopolysaccharide (155). In a 2018 study, our group contributed to test the anti-inflammatory effects of tofacitinib in preclinical models of psoriasis. In detail, we evaluated the immunomodulatory effects of tofacitinib on epidermal keratinocytes activated by IFN-γ, IL-22, TNF-α or IL-17. We demonstrated that, similarly to full-length SOCS1 or SOCS3 in keratinocyte clones (82), tofacitinib suppressed expression of IFN-γ-dependent inflammatory genes, and restored normal differentiative programs altered by IL-22 in psoriatic keratinocytes, whereas it did not influence TNF-α- or IL-17-induced molecular cascades. Of note, tofacitinib strongly ameliorated the psoriasiform phenotype of an imiquimod-induced murine model of psoriasis by breaking the inflammatory cross-talks between stromal resident cells and infiltrating immune cells (156) (**Figure 3**).

## 5 Advances in JAKs inhibitors use in dermatological inflammatory diseases

Currently, several JAK inhibitors are employed in dermatology, and others are being investigated for skin diseases like alopecia areata, psoriasis, and vitiligo.

### 5.1 Atopic dermatitis

The most of FDA-approved JAK inhibitors are currently employed for AD management. AD is an immune-mediated skin disease, characterized by an acute phase occurring within the first 72 h after lesion onset, determined by Th2-driven immune responses with a consequent upregulation of the Th22 axis (157). Thereafter, a chronic phase manifests with continued increase in Th2 and Th22 infiltration and new upregulation of Th1 responses (158). A variety of Th2-related cytokines, in particular IL-4 and IL-13, are involved in the pathogenesis of AD. Other than inhibiting the expression of anti-microbial peptides, IL-4 and IL-13 suppress the expression of filaggrin (FLG), resulting in skin barrier dysfunction. Furthermore, IL-4 and IL-13, together with IL-31 and thymic stromal lymphopoietin (TSLP) are involved in pruritus, whereas Th2-released IL-5 activates eosinophils. Finally, Th22-released IL-22 drives keratinocyte proliferation in the chronic phase (96) (**Figure 3**).

All these cytokines bind to specific receptors and activate specific arrays of JAKs and STATs pathways. Since JAK1 is involved in most of the signaling pathways of those key cytokines for AD (159), inhibition of JAK1 leads to improvement of AD. JAK inhibitors inhibit a wider range of intracellular molecular cascades activated by AD-related cytokines. However, JAK1 associates to receptors of AD-unrelated cytokines or growth factors that contribute to homeostasis and the immune response (160). For instance, IFN-α plays an important role in the innate immune response, and its receptor harbors JAK1 and TYK2, whereas IFN-γ receptor harbors JAK1 and JAK2. Of note, GM-CSF and erythropoietin are involved in erythropoiesis, myelopoiesis, and platelet production, and their receptors harbor JAK2. Therefore, strong inhibition of JAK1 and JAK2 could cause adverse events in addition to amelioration of AD (**Figure 3**). Upadacitinib, a selective oral JAK1 inhibitor, was approved by the US Food and Drug Administration (FDA) and European Medicines Agency (EMA) for the treatment of rheumatoid arthritis in 2019. Currently, upadacitinib is also approved for atopic dermatitis, psoriatic arthritis, ankylosing spondylitis, and gastrointestinal diseases (161). Indeed, upadacitinib exhibited > 40-, 130-, and 190-fold greater selectivity for JAK1 versus JAK2, JAK3, and TYK2, respectively, in cellular assays (162).

As for upadacitinib, abrocitinib is a selective oral JAK1 inhibitor recently FDA-approved for patients ≥ 18 years old with moderate-to-severe AD (163), whereas baricitinib, recently approved in Europe and Japan for the treatment of AD in adult patients (164), is an oral inhibitor of JAK1 and JAK2 with modest activity against JAK3 and TYK2. Finally, ruxolitinib, a JAK1/JAK2 inhibitor, has been recently approved for the topical treatment of AD (165) (**Figure 3**).

Of note, although these three JAK inhibitors have different selectivity, increased dose of their administration can inhibit other JAKs. For instance, although upadacitinib is a JAK1-selective inhibitor, the incidence of anemia, one of the adverse effects of JAK2 inhibition, was higher in patients treated with 30 mg of upadacitinib than in those treated with 15 mg of upadacitinib or placebo during 16 weeks (166). Furthermore, the degree of inhibition is also important in understanding the differences in efficacy and side effects among JAK inhibitors. For example, although baricitinib inhibits both JAK2 and JAK1, its degree of inhibition is moderate, thus having mild efficacy with tolerable safety. In particular, severe anemia, one of the possible adverse effects caused by JAK2 inhibition, was not observed in clinical trials of baricitinib (167) (**Figure 3**).

### 5.2 Psoriasis

Psoriasis is a chronic inflammatory skin disease, affecting up to 3% of the worldwide population (88). Disease pathogenesis results from sustained innate and adaptive immune responses that lead to uncontrolled keratinocyte proliferation and dysfunctional differentiation. The activation of plasmacytoid dendritic cells (pDC) by LL37/cathelicidin released by damaged keratinocytes and complexed with self-genetic material is considered as the triggering event for the development of the psoriatic plaque. pDC thus produce type I IFN promoting myeloid dendritic cell phenotypic maturation, thus leading to Th1 and Th17 cell activation.

Several cytokines have been found to play a key role in the development and maintenance of the inflammatory processes behind psoriatic lesions. In particular, the IL-23/Th17 axis is believed to play a central role (88). Indeed, IL-23 drives the differentiation and proliferation of Th17 cells, which produce high levels of IL-17 and IL-22, causing proliferation of epidermal keratinocytes and impairing their terminal differentiation. Psoriasis has several extracutaneous manifestations, such as inflammatory articular and entheseal involvement, leading to PsA. Recently, tofacitinib and upadacitinib were added to the therapeutic armamentarium for treating PsA (168). In September 2022, deucravacitinib, a selective TYK2 inhibitor, has been approved for the treatment of moderate-to-severe psoriasis (169). Its unique mechanism determines greater selectivity and a reduced risk of adverse events (**Figure 3**).

### 5.3 Alopecia areata

Alopecia areata (AA) is a CD8^+^ T cell-mediated autoimmune disease, characterized by inflammatory cell infiltration around lesion follicles, that affects hair follicles (170, 171). In normal conditions, the anagen hair bulb is preserved by complex immune mechanisms. During the anagen phase, the follicle is protected by its low levels of MHC protein and β2 microglobulin expression, which reduce self-antigen presentation and prevent T-cell activation and proliferation. In AA, cytokines such as IFN-γ and common gamma chain cytokines (IL-2, IL-7 and IL-15), as well as inflammatory NKG2D^+^ CD8^+^ T cells and CD4^+^ T cells predominate. These T cells activate IFN-γ, creating a positive feedback loop, which leads to the premature end of the anagen phase, with entry in catagen phase, which is characterized by apoptosis of the hair cells (172). Tofacitinib, a pan-JAK inhibitor, has been used in the off-label management of AA, resulting to be effective in the treatment of adults and adolescents with AA (173). However, being a pan-JAK inhibitor, tofacitinib may be associated with adverse events (174) (**Figure 3**).

In June 2022, baricitinib has been approved from FDA for AA (175). Numerous other JAK inhibitors are now being studied in AA management. Oral JAK inhibitors seem to be superior to topical JAK inhibitors but a role for topical JAK inhibitors in the management of some aspects of AA treatment (eyebrows, eyelashes, pediatric AA) cannot be excluded.

Although JAK inhibitors are among the most promising therapeutics for AA, the long-term safety and efficacy of JAK inhibitors need to be deepened (**Figure 3**).

## 6 Conclusions and possible future applications

Understanding the functions and roles of SOCS1 and SOCS3 is essential for appreciating their contributions to immune regulation and their implications in various diseases. Their study has opened avenues for important therapeutic interventions, culminating in the development of the current JAK inhibitors, aimed at modulating immune responses in specific conditions, including inflammatory immune-mediated skin diseases. The field of JAK inhibitors is dynamic, with ongoing research contributing to a deeper understanding of their mechanisms and potential applications.

For example, JAK/STAT pathway inhibition can be considered as a promising target for the treatment of vitiligo, a complex skin depigmentation disease that involves multiple pathogenetic mechanisms, including genetic predisposition, oxidative mechanisms and environmental factors, which culminate in the destruction of melanocytes by CD8^+^ cytotoxic T cells (176). IFN-γ-induced signature has been observed in human skin with vitiligo, with the upregulation of cytokines CXCL9 and CXCL10 via JAK1/2. These cytokines contribute to the recruitment of cytotoxic T lymphocytes responsible for melanocyte destruction (177). This is the reason why inhibition of JAK proteins can be considered an effective therapeutic strategy for the treatment of vitiligo (178). Consistently, topical ruxolitinib exhibited efficacy for re-pigmentation in face vitiligo in a preliminary open-label study (179). Currently, there is an ongoing phase-2 study in which patients received a combination therapy of baricitinib and phototherapy (180).

In contrast, more studies are highly needed to test the validity of JAK inhibitors for hidradenitis suppurativa, a chronic, inflammatory, recurrent, and debilitating skin disease of the hair follicles characterized by inflammatory, painful, deep-rooted lesions. There is only one published clinical trial in the literature (JAK1 inhibitor INCB054707), a real-life study with 15 patients up to week 24 with upadacitinib and a case report showing the successful use of tofacitinib (181). Conversely, there are several ongoing clinical trials.

In conclusion, more efforts aimed at deepening the role of SOCS1 and SOCS3 in “treatment orphan” inflammatory or autoimmune skin diseases and improving efficiency and selectivity of JAK inhibitors will permit to advance treatment options for individuals suffering from debilitating skin conditions.

## Author contributions

MM: Data curation, Formal analysis, Visualization, Writing – original draft. SM: Conceptualization, Supervision, Visualization, Writing – original draft. CA: Conceptualization, Supervision, Writing – original draft.
